# Broadband Onset Inhibition Can Suppress Spectral Splatter in the Auditory Brainstem

**DOI:** 10.1371/journal.pone.0126500

**Published:** 2015-05-15

**Authors:** Martin J. Spencer, David A. X. Nayagam, Janine C. Clarey, Antonio G. Paolini, Hamish Meffin, Anthony N. Burkitt, David B. Grayden

**Affiliations:** 1 NeuroEngineering Laboratory, Department of Electrical and Electronic Engineering, University of Melbourne, Melbourne, Australia; 2 National ICT Australia, Department of Electrical and Electronic Engineering, University of Melbourne, Melbourne, Australia; 3 Centre for Neural Engineering, University of Melbourne, Melbourne, Australia; 4 Bionics Institute, Melbourne, Australia; 5 Department of Pathology, University of Melbourne, Melbourne, Australia; 6 Health Innovations Research Institute, RMIT University, Melbourne, Australia; McGill University, CANADA

## Abstract

*In vivo* intracellular responses to auditory stimuli revealed that, in a particular population of cells of the ventral nucleus of the lateral lemniscus (VNLL) of rats, fast inhibition occurred before the first action potential. These experimental data were used to constrain a leaky integrate-and-fire (LIF) model of the neurons in this circuit. The post-synaptic potentials of the VNLL cell population were characterized using a method of triggered averaging. Analysis suggested that these inhibited VNLL cells produce action potentials in response to a particular magnitude of the rate of change of their membrane potential. The LIF model was modified to incorporate the VNLL cells’ distinctive action potential production mechanism. The model was used to explore the response of the population of VNLL cells to simple speech-like sounds. These sounds consisted of a simple tone modulated by a saw tooth with exponential decays, similar to glottal pulses that are the repeated impulses seen in vocalizations. It was found that the harmonic component of the sound was enhanced in the VNLL cell population when compared to a population of auditory nerve fibers. This was because the broadband onset noise, also termed spectral splatter, was suppressed by the fast onset inhibition. This mechanism has the potential to greatly improve the clarity of the representation of the harmonic content of certain kinds of natural sounds.

## Introduction

The auditory brainstem receives input from the auditory nerve, and provides projections mainly to the auditory thalamus, which in turn projects to the cortex. However, the auditory brainstem is not simply a relay. Nuclei of the brainstem are involved in processing behaviorally important sound cues [1]. Information in the auditory nerve is partly carried by the relative timing of action potentials, and these sub-millisecond cues are most accurately decoded early in the sensory pathway [2, 3]. The auditory brainstem predominantly consists of circuits of neurons that have low membrane time constants, capable of decoding the temporal information that is intrinsic to sound stimuli [4].

A characteristic of some neurons within the auditory brainstem is that of onset inhibition [5]. This is a brief hyperpolarization that precedes the first action potential and has been described in the inferior colliculus [6, 7, 5, 8], the ventral nucleus of the lateral lemniscus [5], and the cochlear nucleus [9]. *In vivo* observations of this effect have previously led to a number of conjectures:

- Fast inhibition in T-stellate cells of rats provided by D-stellate cells, was shown to postpone spikes that were coincident with the inhibition [9, 10]. This may mean that the first spikes in a population of neurons become more temporally aligned, assisting in the lateral integration of the information carried by these spikes.
- Onset inhibition may act as an event reference, increasing the information content of first spike latency [5, 11]. The rebound from inhibition occurs with a particular delay, leading to a spike only if the excitatory input coincides with the timing of this rebound. If the excitatory input does not coincide with this delay then the spike is not produced. This process would create a sensitivity to first spike latency, a property that may represent some important features of sound, such as intensity.
- Onset inhibition may create direction sensitivity for frequency sweeps [12, 2]. This hypothesis also suggests that rebound from the inhibition makes a neuron sensitive to first-spike delay. Combining this with lateral synaptic connections may create frequency sweep direction selectivity.
- Onset inhibition may form a component of a mechanism that is sensitive to the duration of brief sounds [13]. The onset inhibition prevents the neuron from firing for some short period of time at the beginning of a sound. This means that only sounds of a certain duration would create activity in that particular neuron.

In this investigation, we hypothesize that onset inhibition assists in the suppression of broadband spectral splatter. This spectral platter, which occurs at the beginning of any sound with a sharp onset, contains very little information about the harmonic content of the sounds. The harmonic component of the sound stimulus could become more prominent if this element of the stimulus is suppressed. This suppression process may be particularly effective when the sound is made up of a stream of very brief harmonic components, such as those that occur in speech.

Most of the conjectures above are not mutually exclusive and most are specific to particular regions of the auditory brainstem. In particular, the idea that the inhibition suppresses the first spike does not necessarily contradict the possibility that the inhibition could act instead to delay the first spikes in other circumstances [5] or in a separate neural circuit.

In this investigation, our hypothesis was tested by developing a model of a cellular microcircuit in the ventral nucleus of the lateral lemniscus (VNLL). It has been previously postulated [5] that the inhibition in this region is provided by inhibitory interneurons that are driven by octopus cells of the posterior ventral cochlear nucleus, which are known to project to the VNLL [14]. The hypothesis was addressed by obtaining and analyzing experimental intracellular *in vivo* data from the VNLL of rats. A computational model of the VNLL circuit was established and speech-like sounds were used as the stimuli. It was possible to create a population of model VNLL cells and observe their collective response to the stimuli. By using a model, it was possible to manipulate the delays present in the circuit and more clearly demonstrate the interaction between excitation and inhibition.

The aim of the project was to discover a functional role for the onset inhibition observed in the data. Using the model it was found that this inhibition could suppress the noise that occurs at the start of sharp onset sounds, improving the representation of the harmonic components of sound.

## Methods

### Ethics Statement

All procedures were approved by the Royal Victorian Eye and Ear Hospital Animal Research Ethics Committee (project approval codes 95/037 and 04/104A).

Twenty-six male Hooded Wister rats were anesthetized using intraperitoneal aqueous urethane (20% wt/vol, dose: 2.6 g/kg; Sigma, Sydney, Australia); supplementary doses were administered when necessary.

### Experimental Methods

A brief description of the experimental procedures is provided here. It is a summary of that provided in previous studies [5, 15]. Quartz glass micropipettes were used to record intracellular neural responses (1.0 mm OD, 0.7 mm ID, Sutter Instruments, Novato, CA) and had impedances between 30 MΩ and 70 MΩ. Cell impalements were signaled by a sudden and stable drop of more than 25 mV in the DC level as well as the presence of synaptic or large action potentials (> 15 mV). Intracellular recordings typically lasted 2 min (maximum: 30 min). Membrane potential records were stored at a sampling rate of 20 kHz or 40 kHz.

Acoustic stimuli were synthesized digitally. Transducers were coupled to the end of each hollow earbar. The acoustic system was calibrated using a Brüel and Kjær measuring amplifier (type 2606, Brüel and Kjær, Nærum, Denmark). Calibration methods allowed acoustic input to be measured in dB sound pressure level (SPL; referenced to 20 *μ* Pa) and confirmed that the response was adequately uniform across the relevant bandwidth.

The stimulus was 80 dB white noise of 50 ms duration with 5 ms rise-fall times. Stimuli were presented to both ears with a 500 ms repetition interval; left and right signals were offset by 200 ms.

Fifty-six of the recorded cells were located in the VNLL (histologically verified). Of these, eight cells showed inhibition before the first action potential, a transient effect that lasted only the few milliseconds [5]. These 8 cells and their response to the noise stimulus were used as the basis for this study. While this is a small sample size, these cells represent a high quality dataset because they are both *in vivo* and intracellular measurements. All were histologically verified to be from the VNLL and not fibers traversing the lateral lemniscus. This verification is important in the lateral lemniscus where, in extracellular measurements, fibers traversing the complex are indistinguishable from local cells.

### Computational Methods

#### Characterization of the Post-Synaptic Potentials

The shapes of the post-synaptic potentials (PSPs), both excitatory and inhibitory, were extracted from the data using a method similar to spike triggered averaging [16]. For each cell’s data-set, the spontaneous activity (no stimulus) was used. A simple moving average was calculated using a window of 8 ms duration as a low pass filter. A pre-defined offset from the moving average was used as a trigger level for the raw data. The amount of offset used was chosen to be larger than the observed magnitude of the noise present in the data and smaller than the size of the observed PSPs. The offset was larger for inhibitory PSPs (IPSPs) because their magnitude was greater. The EPSPs were not significantly larger than the high frequency noise present in the data so a smaller offset was used. It is likely that some small EPSPs that were obscured by high frequency noise were not included and, therefore, the measured EPSP amplitude would be somewhat larger than the true mean value.

When the raw data exceeded the level of the offset moving average, the data 2 ms preceding and 10 ms following that point was accumulated in an averaged record. The MATLAB (Mathworks 2013) method ‘fminsearch’ was then used to fit five parameters to the resulting curve, *τ*_grow_, *τ*_decay_, $\hat{g}$, *t*_*onset*_, and *λ*, in a function describing the membrane voltage:

$$\begin{array}{c} y\left(t\right)=\hat{g}\times \left\{e^{-\left(t-t_{onset}\right)/\tau_{\text{grow}}}-e^{-\left(t-t_{onset}\right)/\tau_{\text{decay}}}\right\}\times H\left(t-t_{onset}\right)+\lambda t, \end{array}$$ (1)

where *y*(*t*) is the membrane voltage and *t* is time, where *t* = 0 is the start of the record arbitrarily set to be 2 ms before the trigger to allow sufficient time to capture the full PSP. The growth and decay time constants of the PSP are *τ*_grow_ and *τ*_decay_, respectively and the amplitude is $\hat{g}$. The onset time of the PSP is *t*_*onset*_. The Heaviside function, *H*(*t*), forces zero value before time *t*_*onset*_. The final portion of the function, *λt*, is included to provide simple compensation for a tendency of the membrane potential to gradually linearly increase during the experiment. This increase was due to the gradual death of the cell and must be included in the fit-function so that it does not influence the optimization of the other parameters.

#### Model Structure and Topology

**Fig 1:**
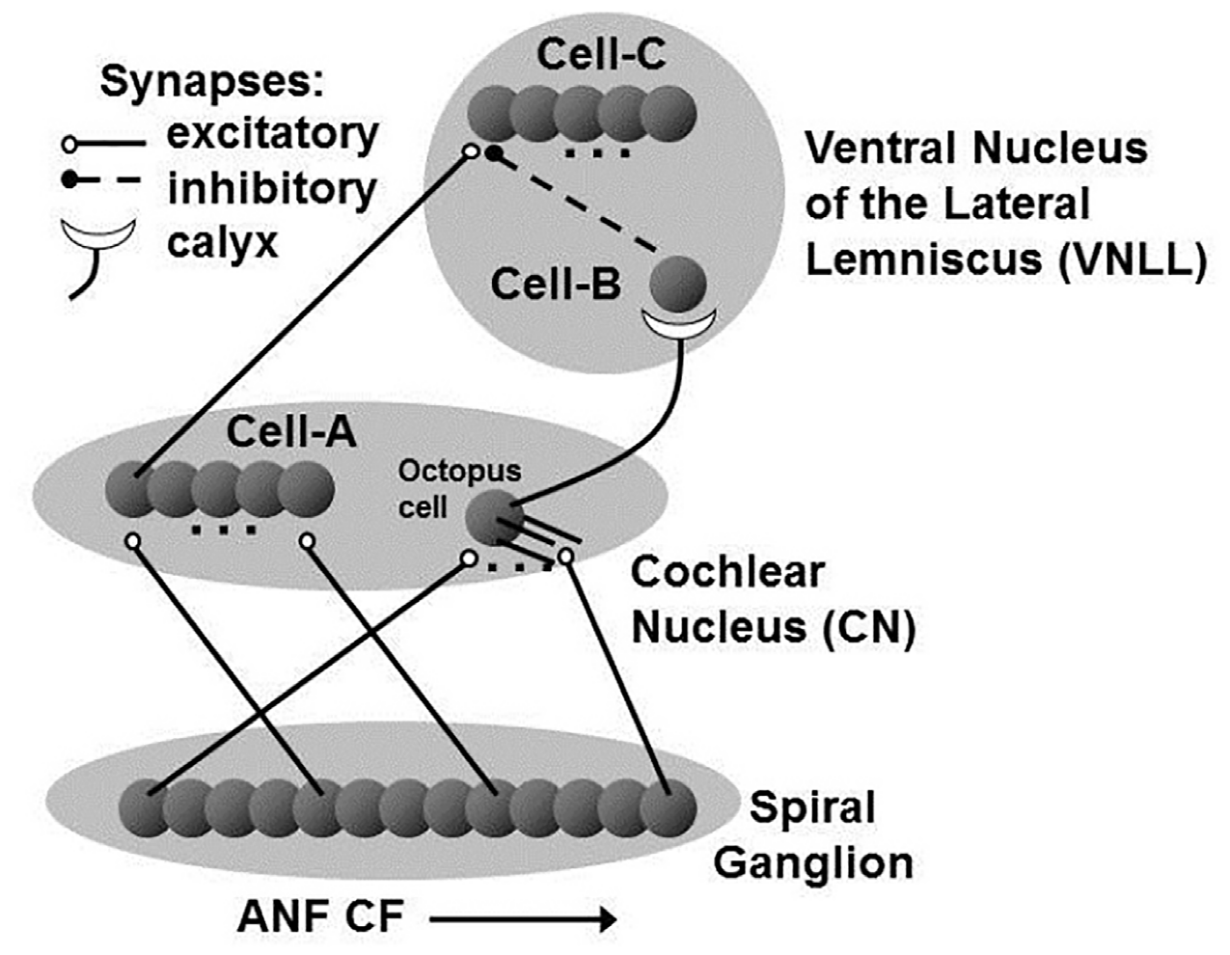
The neuronal circuit investigated in this study. Auditory nerve fibers (ANFs) provide input to two different cochlear nucleus (CN) cell types. Primary-like cells receive input from a single ANF representing a narrow range of frequencies, and octopus cells receive input from a large number (> 60) of auditory nerve fibers representing a broad range of frequencies. The output from these cells re-converge at the VNLL Cell-C neurons with excitatory input from the primary-like CN Cell (Cell-A) and strong onset inhibition from the Cell-B neuron within the VNLL, which in turn receives its input from the CN octopus cell via a secure calyx-like synapse. It is the representation of harmonic information in Cell-C neurons that is the topic of this investigation.

As illustrated in Fig 1, single modeled octopus cell was provided with excitatory input from 350 model ANFs. Excitatory input to the 194 model Cell-C cells was provided by 200 model Cell-A cells (modeled as ANFs), 6 excitatory inputs to each Cell-C neuron. The model was developed using MATLAB. The time step used in the model was 10 *μs*.

Each neuron of the Cell-A population within the cochlear nucleus (CN) receives excitatory input from a single ANF and provides excitatory input to a Cell-C neuron within the VNLL. The Cell-A neuron was modeled as a primary-like neuron, something that reduced the complexity of the model. There is a transformation of the spike-code between ANFs and primary-like CN neurons; in particular, inhibition sharpens the frequency tuning curve and ANF convergence leads to reduced spike jitter. However, if included, it is likely that these components would only make the results of this investigation more robust. An experimental contradiction to the use of primary-like input would not necessarily lead to failure of the hypothesis and a test of this assumption is an area for further work.

Effectively, Cell-A neurons were modeled as a simple relay with a time delay to compensate for the traveling wave delay introduced by the mechanics of the cochlea. This traveling wave delay leads to longer first-spike delays for lower CF ANFs. Therefore, Cell-A neurons with the lowest CFs were given no additional delay. Cell-A neurons with higher CFs were equipped with appropriate delays that brought the spiking activity across frequencies into temporal alignment.

Octopus cells, which receive excitatory input from auditory nerve fibers representing a broad range of frequencies, project to Cell-B neurons in the VNLL. They were modeled using a leaky integrate-and-fire (LIF) model, described in more detail below. LIF models were chosen for the cells in the circuit because they were able to capture the dynamics of the circuit relevant to the testing of the proposed hypothesis with a small number of parameters. Although octopus cells were not the main focus of this study, the overall circuit critically depends on their behavior, and so care was taken to ensure that the octopus cell model matched the experimental data for this cell as explained in the results section.

Cell-B neurons within the VNLL receive excitatory input from octopus cells in the CN via a large, Calyx-like synapse [17] and project to cells in the Cell-C neuron population. Due to the robust and secure nature of this synapse, these cells are expected to provide 1:1 input/output signaling of action potentials. In this study, the Cell-B neuron was modeled as a simple relay combined with a delay, effectively inverting the octopus cells, such that they provide inhibition instead of excitation.

Each cell-C neuron within the VNLL receives excitatory input from Cell-A neurons and inhibitory input from a Cell-B neuron. Like the octopus cell, they are modeled using a LIF approach. The description, constraint, and analysis of this Cell-C neuron model forms the majority of this study.

The parameters of the cells and the circuit were obtained from experimental data that were then optimized using a very simple search algorithm in which parameters were modified randomly and models that produced better results were selected. An initial set of model parameters was chosen and then a number of “children” were instantiated by introducing random variation to each parameter. Each of the “child” models was run, and their quality ranked based on a metric *γ*. The model that produced the highest value for the metric became the basis for the next set of “child” models. Although the metric tended to increase over time, there was nothing to stop the metric from decreasing from one generation to the next due to the influence of the randomization.

#### Auditory Periphery Model

Input to the Cell-A neuron and octopus cell was provided using an auditory periphery simulation [18, 19, 20] the particular model used is realistic in recreating the correct spiking behavior of the auditory nerve through adjustments to the outer ear filter and traveling wave delay. In those publications, the authors explain that they used a range of sounds, including tones, clicks, and speech sounds during validation of this model. The model has a middle ear filter that gives realistic responses to broadband signals. It has realistic cochlear tuning characteristics and produces appropriate jitter statistics of phase-locked spike times. It also provides physiologically realistic group delay and phase properties as a function of sound pressure level and location on the basilar membrane.

#### Brainstem Neuron Models

The LIF model of the octopus cell and Cell-C neuron used a single membrane voltage variable (*V*_m_) that evolved according to an equation that captures the sub-threshold dynamics of the system in response to the transient influences of synaptic input,

$$\begin{array}{c} C_{m}\frac{dV_{m}}{dt}=g_{\text{leak}}\left(V_{\text{rev}}-V_{m}\right)+g_{\text{ex}}\left(E_{\text{ex}}-V_{m}\right)+g_{\text{in}}\left(E_{\text{in}}-V_{m}\right), \end{array}$$ (2)

where *C*_*m*_ is the membrane capacitance, *g*_leak_ is the membrane’s leak conductance, *V*_rev_ is the reversal potential of the leak conductance, *g*_ex_ is the excitatory synapse membrane conductance, *E*_ex_ is the excitatory reversal potential, *g*_in_ is the inhibitory synapse membrane conductance, and *E*_in_ is the inhibitory reversal potential.

The conductances are given by:

$$\begin{array}{ccc} g_{\text{ex}} & = & {\hat{g}}_{\text{ex}}.\left\{e^{-t/\tau_{\text{ex},\text{grow}}}-e^{-t/\tau_{\text{ex},\text{decay}}}\right\}\\ g_{\text{in}} & = & {\hat{g}}_{\text{in}}.\left\{e^{-t/\tau_{\text{in},\text{grow}}}-e^{-t/\tau_{\text{in},\text{decay}}}\right\}, \end{array}$$ (3)

where *g* is the synaptic strength, $\hat{g}$ controls the amplitude, and *τ*_grow_ and *τ*_decay_ define the rate of growth and decay of the post-synaptic potential (PSP), respectively. Each synapse onto a given cell was assumed to have the same strength. Since this is not the case in a real cell, the assumption was tested by also introducing a distribution of synaptic strengths and quantifying the extent of the detrimental effect upon the behavior of the Cell-C neurons.

In the LIF model, a spike is initiated in one of two ways: (a) when the membrane voltage exceeds a threshold (as in the case of the conventional LIF model), or (b) when the rate of change of the membrane potential exceeds a threshold. In each case, after the spike, the membrane voltage is reset. In the case of the Cell-C neuron, the choice between (a) and (b) was investigated. In the case of octopus cells, the latter case (b) is known to be the true behavior observed experimentally [21, 22]. The rate of change of the membrane voltage is calculated over one time step (10 *μs*), and a spike is initiated when this value exceeds a threshold. This is a simplification of previous detailed modelling work [23].

### Model Development and Data Analysis

The investigation proceeded in four stages. In the first stage, the experimental data from the Cell-C neuron population were analyzed and used to determine the parameters of their EPSPs and IPSPs and the nature of the spiking mechanism. In the second stage, the octopus cell model was established. Published experimental data, including *in vitro* current clamps and *in vivo* responses to sound, were used to constrain the parameters of the model. In the third stage, the experimental data acquired from the Cell-C neuron population were used to constrain the Cell-C neuron model. Finally, a population of modeled Cell-C neurons was created and analyzed. The analysis was only specifically intended to reveal the Cell-C neuron population’s representation of the tonal carrier of a sound with a saw-tooth envelope. The quality of the representation of the carrier was examined with respect to variance in the key parameters of the circuit.

## Results

### Experimental Results

**Fig 2:**
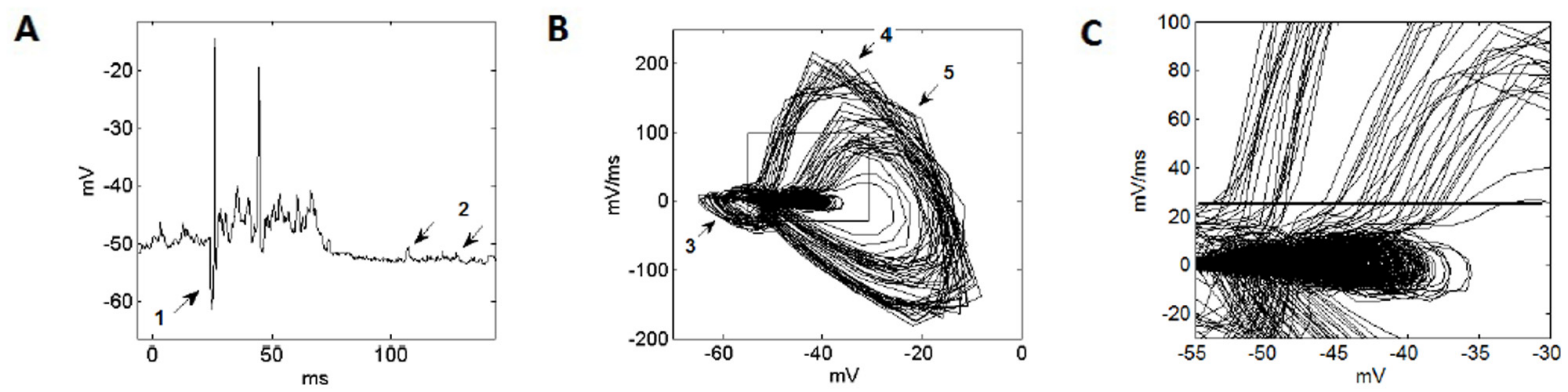
An example of the electrophysiological data from VNLL cells. (A) The membrane voltage in response to the 50 ms noise burst. Arrows indicate the fast inhibition before the first spike (1) and two spontaneous EPSPs (2). (B) A phase plot showing the instantaneous rate of change of the membrane voltage versus the membrane voltage using the 20 instances of the cell’s response to the 50 ms noise data. The arrows indicate the lobes associated with the fast IPSP (3), the action potentials associated with a return from inhibition (4), and action potentials due to subsequent EPSPs (5). The box shows the region expanded and shown in (C) an expanded plot of the region of action potential initiation. The horizontal line is drawn by eye, and shows the apparent action potential initiation point.

White noise at 80 dB, lasting 50 ms with 5 ms ramped onset/offset was used as stimulus in the experimental study. The Cell-C neuron membrane voltage in response to the noise stimuli is shown in Fig 2A. A phase plot (Fig 2B) shows 20 instances of the cell’s response to the noise bursts in a different form. In this plot, the instantaneous rate of change of the membrane voltage, calculated using the difference between subsequent voltage measurements, is plotted as a function of the membrane voltage. A simple 100 ms moving average was used to estimate and the subtract any slow change in the membrane voltage. The quiescent point and spontaneous EPSPs are seen as activity with low rates of change around -50 mV. Induced EPSPs are seen as activity around -40 mV, sometimes resulting in action potentials. The lobes to the left of the plot (3) are the fast onset IPSPs. There are action potentials associated with the return from inhibition (4), and action potentials resulting from subsequent EPSPs (5).

The salient element of the phase plot in Fig 2B is the location of initiation of the action potentials, a region that is shown in more detail in Fig 2C. The plot cannot reveal the underlying causes of action potential initiation, but can show the dynamics of the overall mechanism. This is useful in creating a phenomenological model of the behavior of the neuron. A line is superimposed on Fig 2C delineating the region below which the membrane voltage did not necessarily induce an action potential, and above which action potentials inevitably occur. Although this line may not be precisely horizontal, for the purposes of simplification of the model, it was assumed that it could be modeled as a single value of the membrane voltage gradient. This particular cell appeared to produce a spike at a threshold around 25 mV/ms.

**Fig 3:**
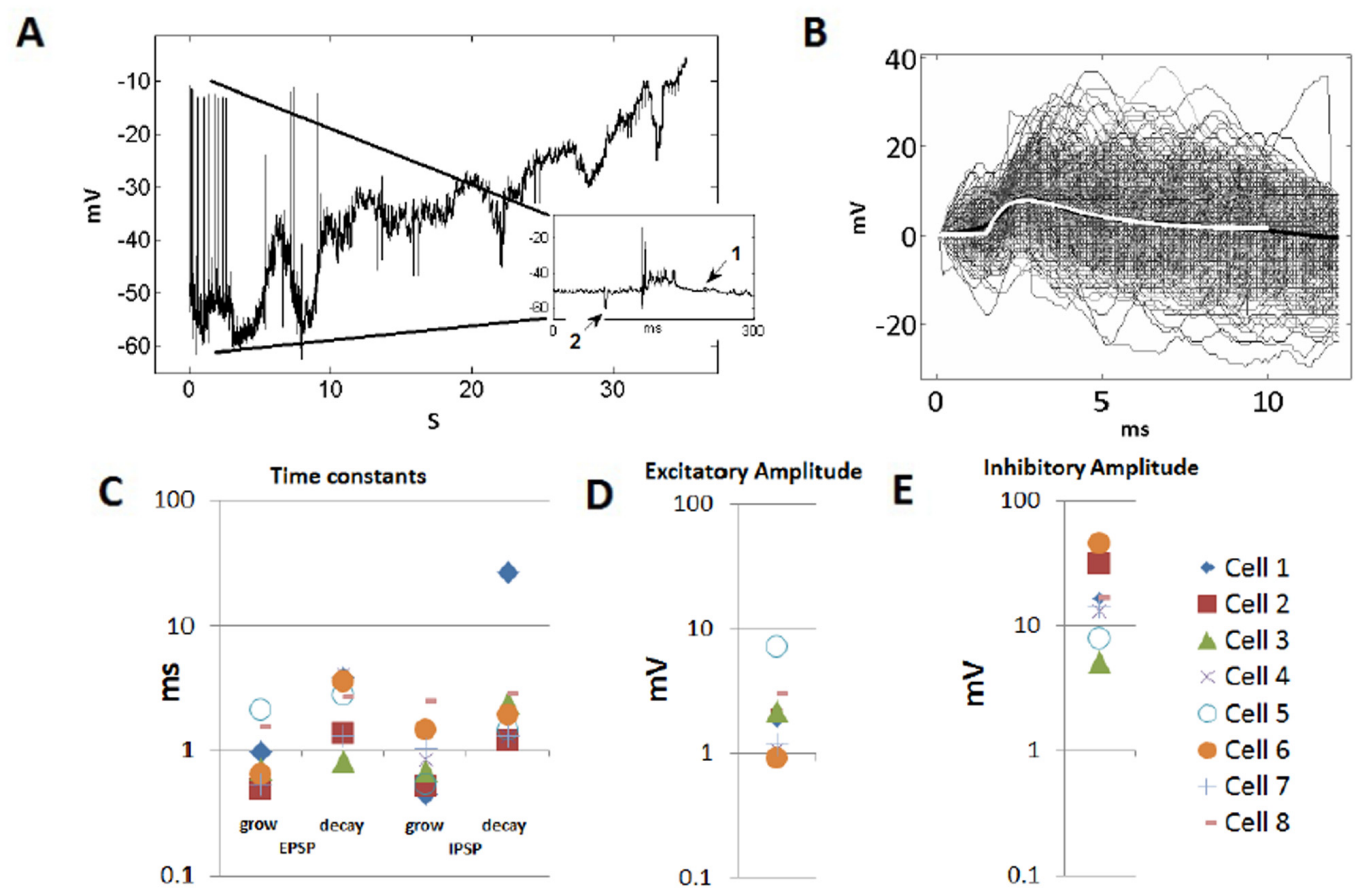
Intracellular recording from a rat VNLL neuron [5]. (A) A Cell-C neuron experimental data set. A large scale is used to show the gradual shift in the cell’s membrane voltage, something that must be compensated in the formula used to fit the EPSPs. In the inset, a magnified portion of the data is shown of approximately 300 ms in duration with spontaneous EPSPs, arrow (1), a spontaneous IPSP, arrow (2), and the cell’s response to the noise stimulus. (B) The triggered snapshot dataset allowing quantification of the EPSP shape. The average is shown as a thick black line and the fit, using Eq 1, is shown with a thick white line. (C) The resulting measured growth and decay time constants of the EPSPs and IPSPs for all eight cells that showed fast onset inhibition. (D) The measured amplitude of the EPSPs. (E) The measured amplitude of the IPSPs.

A portion of raw data from a Cell-C neuron recording is shown in Fig 3A. The data show the membrane voltage in response to a repeated noise stimuli. An example of all of the EPSPs accumulated for one particular cell, as well as the mean, is shown in Fig 3B. Values of the three key parameters, *τ*_grow_, *τ*_decay_, and $\hat{g}$, were evaluated with Eq 1 using the method described. Quantities associated with both the inhibitory and excitatory PSPs for each of the 8 Cell-C neurons are shown in Fig 3C, 3D and 3E. It can be seen that the growth and decay rates of both the EPSPs and IPSPs were of the same order of magnitude. The amplitude of the IPSPs tended to be approximately 10 times the magnitude of the EPSPs.

Rather than taking a mean of the parameters of the eight cells with *in vivo* data, one cell was chosen as representative and was used as the basis of the model Cell-C neuron. This was done because an “average” cell may be unrepresentative of the Cell-C neuron population, while the single cell at least is known to exist in the brainstem with the measured parameters. The particular cell, cell 7 from Fig 3, was chosen because none of its six experimentally determined parameters were the maximum or minimum example of that parameter for the eight cells.

### Constraining the Computational Model

#### Octopus Cell

The parameters of the octopus cell model were based on published experimental data (Table 1). These data informed the choice of the parameters both directly and indirectly.

**Table 1:** Octopus cell model parameters and their values.

*Octopus Cell*	*Value:*	*Reference:*
***Intrinsic Properties***		
Resting potential	-65 mV	Typical arbitrary value
Membrane time constant	300 *μs*	[26, 24]
Spike threshold	10 mV/ms†	[21]
Leak Conductance	7 MΩ*	[24]
Total Dendritic Delay	0.5 ms‡ *	[27, 23]
***Excitatory input***		
Number of afferent ANFs	350	Indirectly constrained
CF of afferent inputs	5.7–20.0 kHz	Indirectly constrained
Time constant of synaptic excitation	1.2 ms	[28] (mice)
Axonal delay from excitatory input neuron	0–0.5 ms*	[27, 23]§
Conductance change per afferent spike	0.87 nS	[28] (mice)
Reversal potential	0 mV*	[28] (mice)

* These parameters were taken directly from the experimental results of the papers cited.

^†^ This rate-of-change was calculated over a single time step (10 *μs*).

^‡^ The total dendritic delay available is 0.5 ms but the individual dendritic delay applied to each synaptic input was dependent on the characteristic frequency (CF) of the cell, with the input spikes from the highest CF ANFs receiving the longest delay.

^§^ Given the results in these papers, the ANF differential first spike latency due to the cochlear traveling wave delay is taken to match and be compensated for by the octopus cell dendritic delay.

Initially, the behavior of an *in vitro* model (without synaptic input) was compared against the experiment to confirm that its behavior resembled that of the real cell. The parameters of this *in vitro* octopus cell model are defined in Table 1. The *in vitro* comparison used simulated injected currents to re-create a previous experimental investigation [24]. The model octopus cell showed the correct, experimentally observed response to the onset of the current steps and pulses with a new action potential in response to each new current pulse or step. A continued constant current stimulus did not result in further action potentials.

With synaptic input provided by the periphery model, the behavior of the model cell could be tested against *in vivo* experimental data. The ANF synaptic input was provided with the frequency range 5.7–20 kHz as defined and described in Table 1. This decision was based on previous work, which showed that the input to octopus cells is likely to come from high frequency ANFs [23]. The particular range was chosen so that the cochlear traveling wave delay was matched and compensated by the dendritic delay.

**Fig 4:**
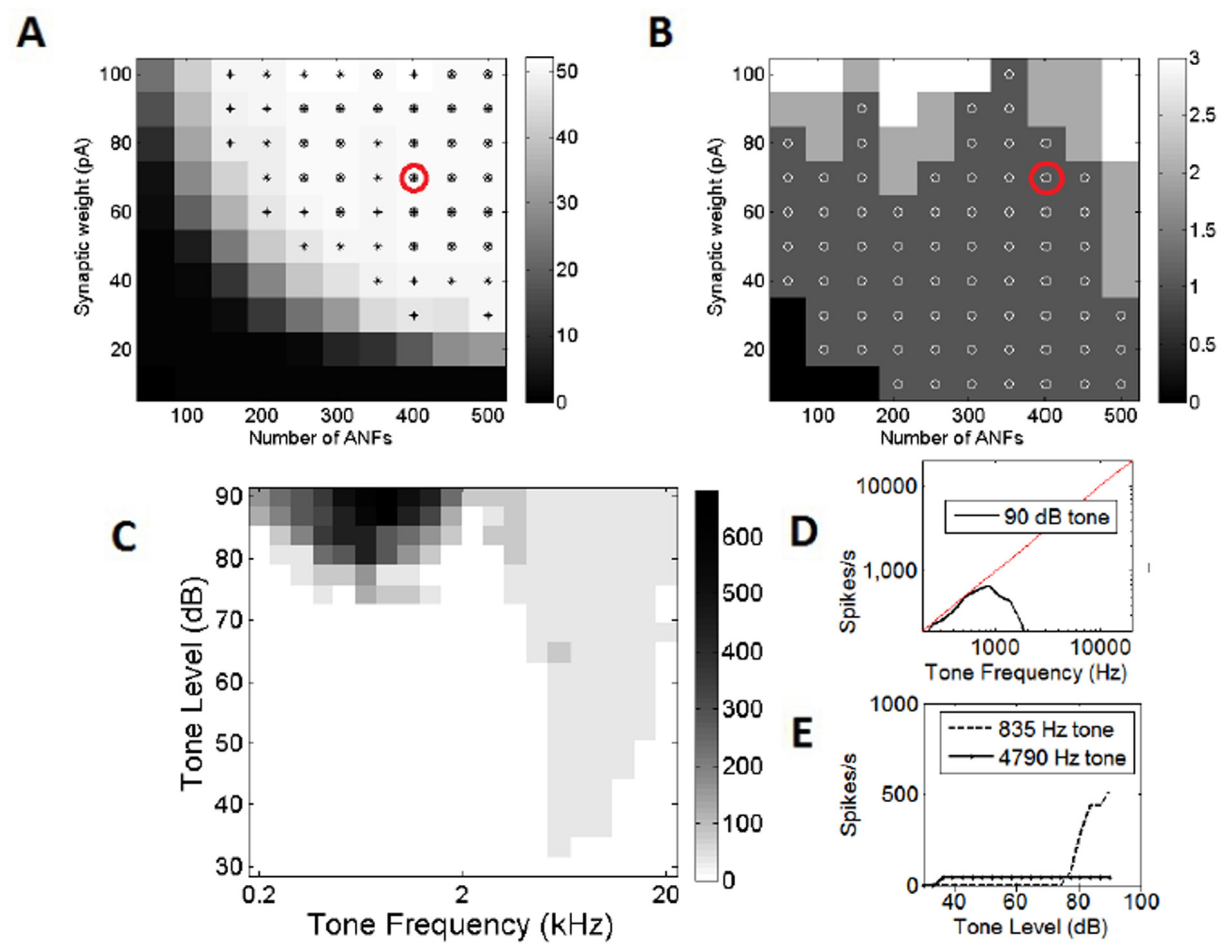
Examination of the total synaptic drive to the octopus cell. (A) The number of spikes of the octopus cell in response to a click train consisting of: fifty-one clicks at 500 Hz. The number of spikes produced by the octopus cell model is recorded as a function of both the number and strength of synaptic inputs to the octopus cells, and is recorded as a grey-scale key to the right of the plots. The crosses show that the number of spikes were between 41 and 61 spikes. The small circles show configurations which produced precisely 51 spikes, the expected result. The red circle shows the final model chosen. (B) A similar analysis, however the stimulus is the noise burst used as the search stimulus in a previous experimental investigation [5]. In this case, the small circles show the instances in which the octopus cell produced one spike. The red circle shows the final model chosen. (C) The number of spikes of the model octopus cell in response to 25 ms input tones of various frequencies and SPLs. (D) The number of spikes in response to a 25 ms tone at 85 dB as a function of tone frequency. The grey line shows the number of spikes expected if the cell responds with perfect entrainment. (E) The number of spikes of the model octopus cell in response to the tones with the frequencies shown as a function of intensity.

Trains of clicks were used as stimuli, the same as those used in a previous experimental investigation [24]. Briefly, the stimuli consisted of 51 clicks at 80 dB, each separated by 2 ms. The number of action potentials produced was recorded as a function of the number of synapses and their strength. It was expected that 51 action potentials would be produced, one for each click sound. The results with a membrane voltage rate of change threshold of 15 mV/ms, are shown in Fig 4A. Similar plots were also produced for model cells with membrane voltage rate of change thresholds of 5, 8, and 12 mV/ms covering the experimentally observed range of 5 mV/ms to 15 mV/ms. Additionally, the 50 ms noise stimulus described above was used and action potentials counted. In this case, it was expected that only one action potential should be produced (Fig 4B). There were a number of configurations that provided the correct response to both the noise stimulus and the click train. The configuration chosen was that with 400 ANF inputs and 70 pA strength per synapse. This configuration occupied the region of parameter space that most reliably produced the correct behavior.

Many *in vivo* experimental studies use frequency-sound pressure level analysis [25, 15], and the same approach was used here (Fig 4C). Tones of 25 ms duration with a 2.5 ms linear rise/fall ramp were used. The sound pressure level and frequency of the tones were varied. It can be seen that the model provided the typical octopus cell frequency response area. At tone frequencies below around 800 Hz, the model reliably entrained (phase-locked to every period of the stimulus tone). With stimulus tones between 1 and 2 kHz, the model phase-locked to the tone stimulus but did not produce an action potential in response to every period. With stimulus tones above 2 kHz, the model octopus cell produced one action potential at the onset of the tone and no further activity.

The cell’s response at 90 dB is shown in more detail (Fig 4D); it can be seen that the cell produced action potentials at a rate that matches the tone’s frequency up to approximately 800 Hz. This is consistent with entrainment. The activity of the cell as a function of sound intensity was checked at the measured characteristic frequency (CF), in this case 4790 Hz, as well as a tone with a frequency of 835 Hz, the approximate frequency at which the octopus cell fires at the highest rate (Fig 4E). It can be seen that with a CF tone, the model octopus cell began to respond at a minimum intensity of 40 dB. With a tone stimulus of 835 Hz, the model cell began to respond at around 60 dB and fire at rates consistent with entrainment at around 85 dB (Fig 4E).

**Fig 5:**
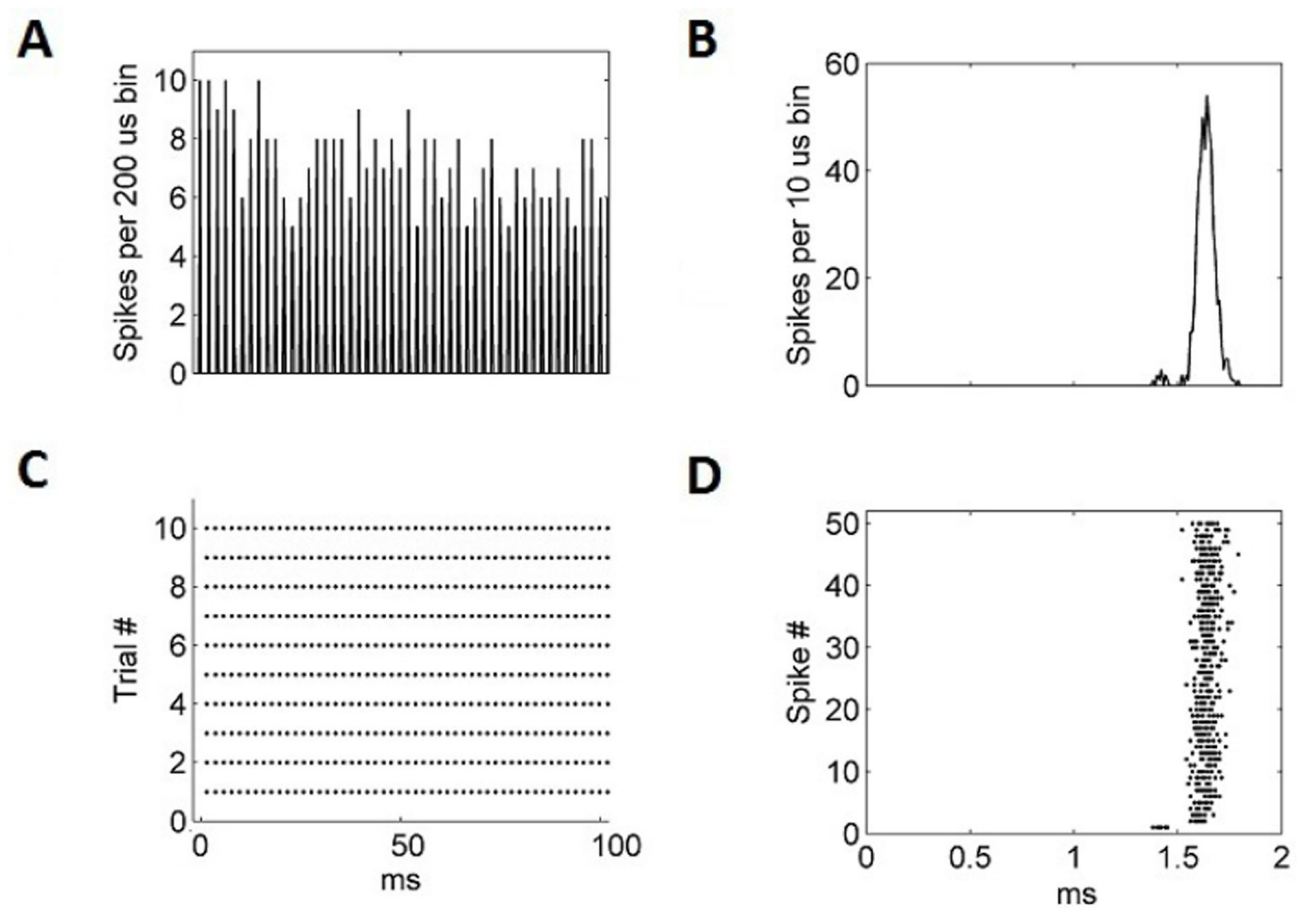
(A) The PSTH of the model octopus cell in response to 10 trials of 51 click stimuli. The clicks were separated by 2 ms and presented at an amplitude of 80 dB. (B) The cumulative histogram of all 510 click stimuli re-framed into a single 2 ms period. (C) The individual spikes over the 10 trials. (D) The timing of each of the 510 spikes, reframed into a single 2 ms window, and shown as a function of spike number (1–51). This figure replicates an experiment carried out in a previous experimental investigation [24].

An experimental analysis used in a previous experimental investigation [1] was also simulated using the model. The click train (51 repetitions) was repeated in 10 trials. The post-stimulus time histogram (PSTH) (Fig 5A) appears to show jitter in spike timing. However, by comparing Fig 5D with experimental data [24], it can be seen that this was actually because the model’s spike response latency gradually increased over the subsequent 100 ms stimulus. After an initial jump in timing from the first to second spike, this gradual latency shift between spikes 2–51, rather than any increase in jitter, is what explains the variation in peak height seen in Fig 5A. This effect was also observed in experimental data [24].

The 2 ms windowed spike latency plot (Fig 5D) shows that the first spike occurred at an earlier time with respect to the stimulus than subsequent spikes. Usefully, our model showed that that this effect is not due to the behavior of the octopus cell. In fact, it is the ANFs that have a first spike that arrives earlier relative to the stimulus than the later spikes. This then causes the octopus cell, which receives input from these fibers, to follow this behavior and this creates the ‘early’ first spike seen in the experiment.

#### Cell-C neuron

As described in the experimental results, a single cell was chosen as the basis for the model Cell-C neuron. A number of parameters remained that were unconstrained and these are labeled in the summary of the model parameters in Table 2. A number of these were set to arbitrary and nominal values and were subsequently tested to ensure that the final function of the model was not sensitive to their precise selection.

**Table 2:** Cell-C neuron model parameters and their values.

*Parameter:*	*Values:*
***Intrinsic Properties***	
Resting potential	-65 mV*
Reset potential	-75 mV *
Membrane capacitance	12 pF *
Leak Conductance	140 nS *
Spike threshold	8 mV/ms
***Excitatory input***	
Number of afferent ANFs	6
Grow time constant (*τ*_grow_)	0.54 ms
Decay time constant (*τ*_decay_)	1.3 ms
Input delay	1.6 ms
Synaptic amplitude ($\hat{g}$)	1.2 mV
***Inhibitory input***	
Number of afferent octopus cell axons	1
Grow time constant (*τ*_grow_)	1.0 ms
Decay time constant (*τ*_decay_)	1.3 ms
Synaptic amplitude ($\hat{g}$)	14 mV
Input delay	1.2 ms
Reversal Potential	-180 mV

* These parameters are not constrained by the experimental data. Their values are simply assumed as typical values. The leak conductance, and random variation in the excitatory synaptic inputs were later tested for their effect on the function of the model.

There is no evidence regarding the precise source of the input to the VNLL cells that we term Cell-C neurons. However, there is evidence that input to the nuclei of the lateral lemniscus comes from the ventral cochlear nucleus [29], an area that contains primary-like neurons. As explained in the methods section, we assume that Cell-C neurons receive input from primary-like cells with high spontaneous rates. This primary-like excitatory input to the Cell-C neuron model was provided by the periphery model. It was also assumed that these inputs represented a narrow range of tonal frequencies. The primary-like Cell-C neuron input cells were spaced evenly in log scale between 2 kHz and 20 kHz.

The number of inputs was constrained by matching the model and experimental response average amplitudes. This was done with data obtained during the period immediately after impalement, whilst the membrane potential remained stable to reduce the influence of cell death. Specifically, the average deviation was calculated with the action potentials removed, over 10 identical instances of the stimulus. The observed average deviation from the initial membrane voltage was around 6 mV. The model’s spike mechanism was removed and the model was run with different numbers of inputs between 3 and 10. It was found that 6 excitatory inputs led to a similar amplitude of response during noise as the typical experimentally observed cell. As a secondary confirmation that this was a reasonable value, the spontaneous PSP rate in the experimental data and the model were compared because it should be expected to be proportional to the number of inputs.

The spontaneous rate of excitatory input in Cell-C neurons is difficult to measure because the EPSPs are small and therefore sometimes obscured by noise, as already described in the methods. However, the spontaneous rate of EPSPs in the model and experiment appeared to be of the same order of magnitude when there were between 5 and 10 excitatory inputs as measured by counting visible individual EPSPs over several noise stimulus cycles.

The spiking mechanism was chosen to depend on the rate of change of the membrane voltage. The decision was based on the observation that the experimental cells appeared to possess such a spike initiation mechanism (Fig 2). The magnitude of the spike threshold was based on the cell’s response to the noise search stimulus. A range of values were tested. With a threshold of 8 mV/ms, the model produced one spike at the beginning of the noise (a rebound response to the decay of the large IPSP). In addition, the noise stimulus occasionally induced further action potentials in the model. These qualities matched the behavior seen in the experimental data. The value of 8 mV/ms is somewhat smaller than the value of 25 mV/ms discovered in Fig 2; however, it is to be expected that there is variation in the properties of different cells.

**Fig 6:**
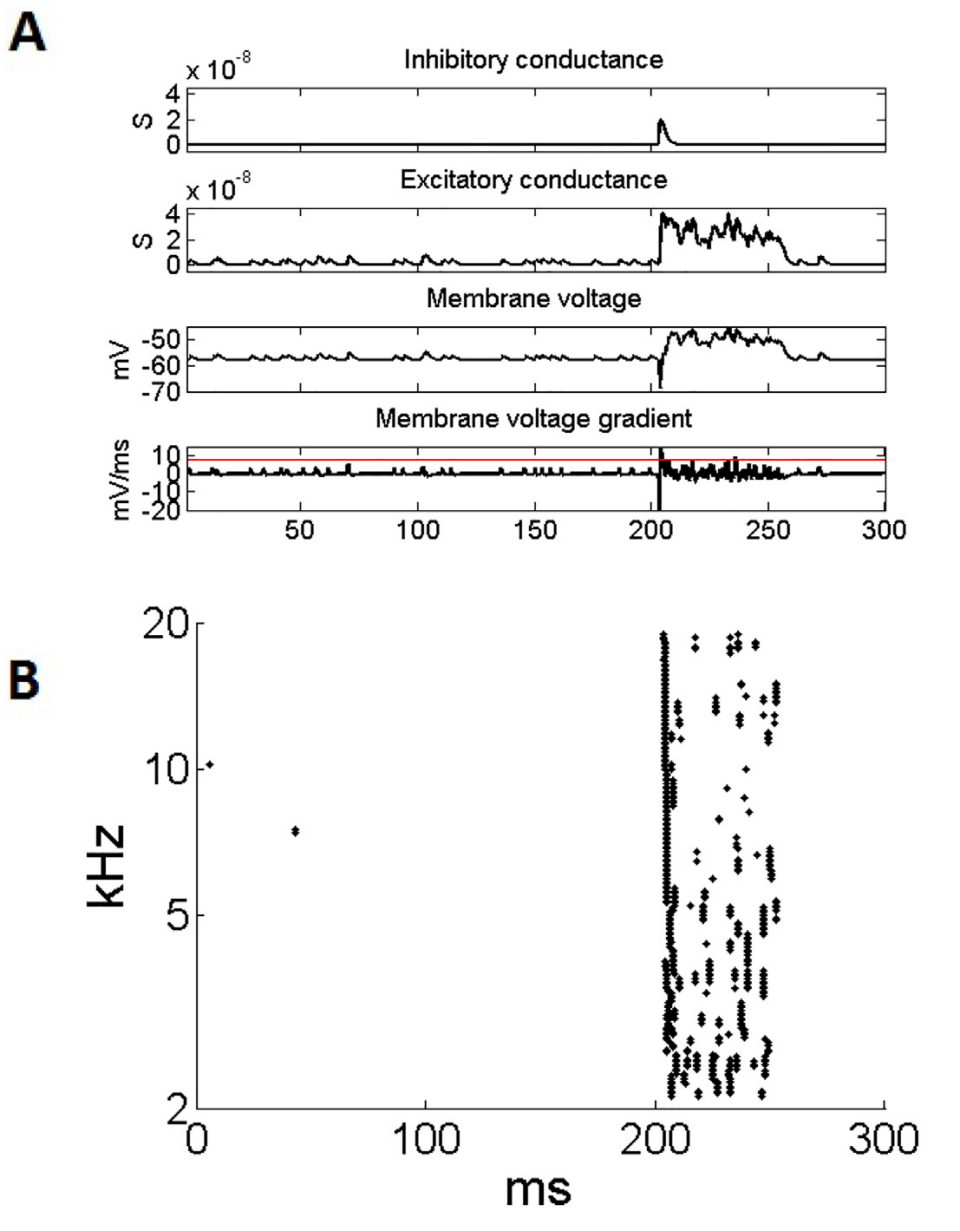
The response of the model Cell-C neuron to the noise search stimulus with an action potential trigger of 8 mV/ms. (A) An individual cell’s response to the noise stimulus. The total inhibitory and excitatory conductances are shown along with the membrane voltage and its gradient. The trigger level (horizontal line) is indicated on the plot of the gradient of the membrane voltage. (B) The population of cells showing their spiking response to the same noise stimulus. The population is made up of Cell-C neuron model cells with a range of CFs.

With this final calibration, the model behavior reflects not just intrinsic properties of the cell, but the behavior of the network and all of its properties. It was therefore possible to compare the behavior of the model to the behavior of the real circuit. The model cell’s behavior was simulated in response to the noise stimulus used in the experiment (Fig 6). The model cell’s membrane voltage in Fig 6A can be compared to the recorded membrane voltage shown in Fig 2A. Note that the effect of action potentials on the membrane voltage is not included in the LIF model, however it can be seen to have a close qualitative match. Of course, with the model, it is also possible to examine the internal dynamics of the cell, and this is partly done in Fig 6A by plotting the contributions from the excitatory and inhibitory currents in the cell. It can be seen that there is an initial inhibition followed by continuous excitation, and the effects of this upon the membrane voltage can be seen to replicate the membrane voltage in the real cell. The spiking activity was also recreated for a population of cells with a range of different CFs in Fig 6B. It can be seen that the Cell-C neuron population responds with a similar pattern, independent of their CF. This can be compared with the data in a previous experimental investigation [5].

### Computational Model Results and the Function of the Neural Circuit

**Fig 7:**
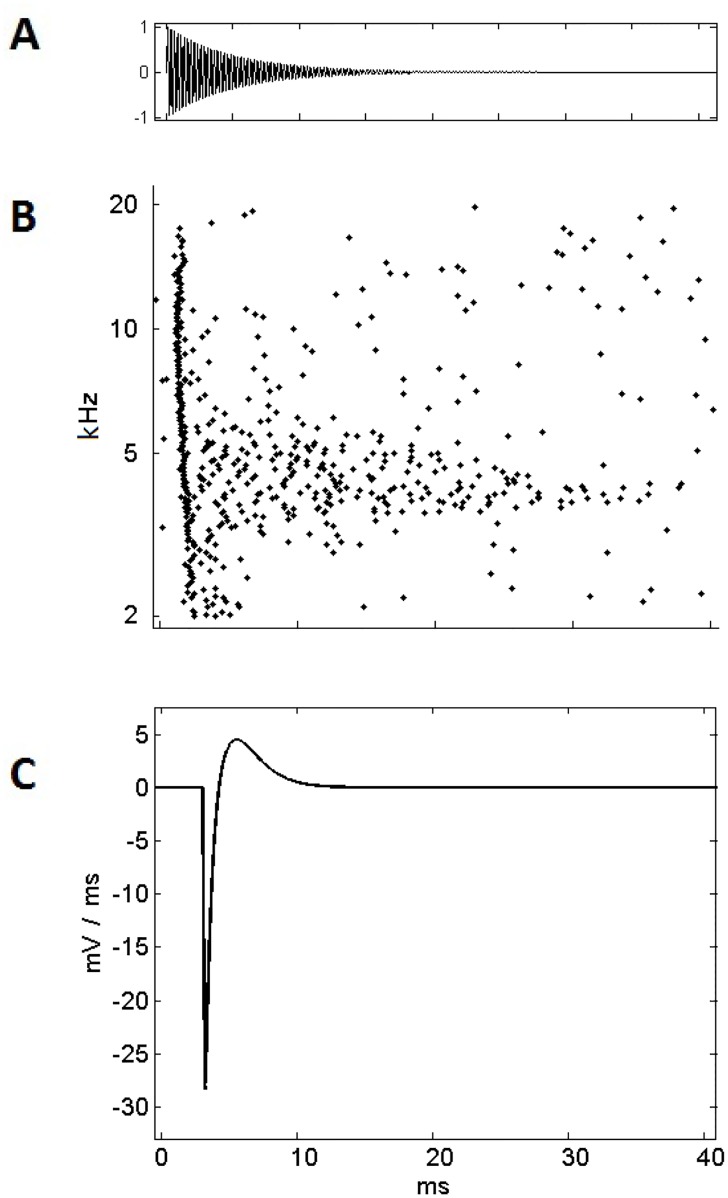
(A) A percussive stimulus consisting of a 65 dB damped tone of 4 kHz. (B) The response of a population of primary-like neurons shows that the tone’s frequency is initially obscured by the spectral splatter at the onset of the sound. The best representation of the tone frequency immediately follows this with greatest activity centered on 4 kHz. (C) The first-derivative of the membrane voltage, under the influence of an IPSP. This represents the IPSP’s effect on the cell’s likelihood of producing an action potential.

The hypothesis that onset inhibition may assist in the suppression of broadband spectral splatter was explored. Fig 7A shows a tone with an instantaneous onset followed by a decay offset that was used as the stimulus. This form of stimulus is characteristic of any sound produced percussively, not uncommon in the natural environment.

It is this kind of sound that is most susceptible to the interfering signal of onset splatter in the auditory nerve and that we propose is better represented by the Cell-C neurons in the VNLL. To understand the mechanism, three observations were made:

First: Fig 7B shows the response to the stimulus from the cells providing excitatory input to the Cell-C neuron. The spectral splatter in the first 1–2 ms results from the sharp sound onset obscuring the harmonic content of the sound, in this case the pure tone at 4 kHz. Following this initial broadband splatter, the tone’s frequency of 4 kHz is represented by the population of fibers, with activity focused on the 4 kHz fibers.

Second: The synchronous broadband activity seen in Fig 7B in the first 1–2 ms of the response to the sound is precisely the activity that triggers action potentials in an octopus cell.

Third: Octopus cells are proposed to provide inhibitory synapses to the Cell-C neurons and Fig 7C shows the first derivative of the membrane voltage of the cell in response to an IPSP. The first derivative was used because we have already observed that the Cell-C neuron produced spikes at a particular *rate of change* of the membrane voltage. It can be seen that the IPSP tended to reduce the cell’s propensity to produce spikes at the immediate onset of the sound, but following this, enhanced the cell’s propensity to spike.

By combining these three observations, it is possible to see that the onset inhibition in Cell-C neurons might provide suppression of the initial onset splatter in the excitatory input. This would leave the response to the underlying harmonic content of the sound, in this case a 4 kHz tone.

**Fig 8:**
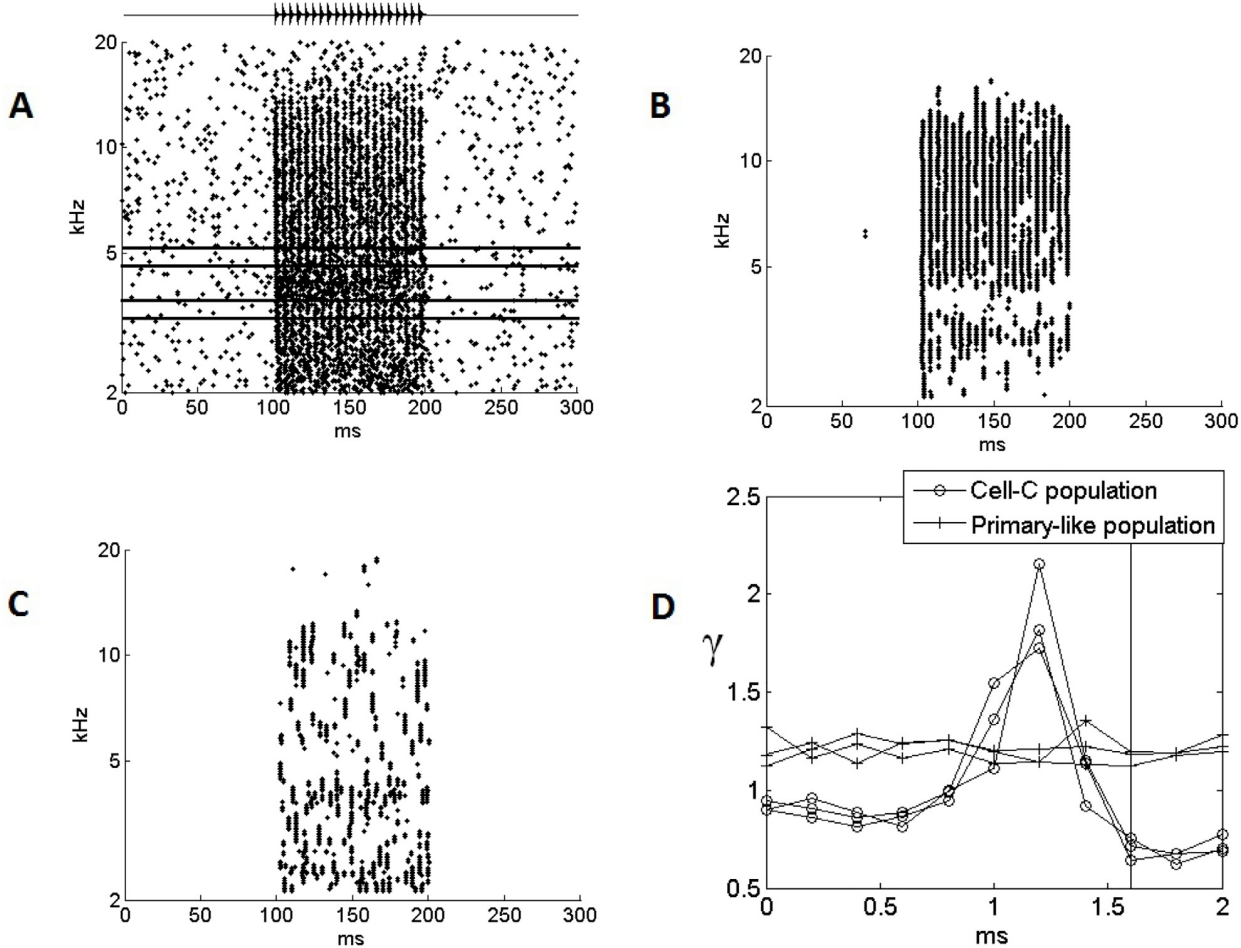
The response of the cell population under the influence of onset inhibition. The behavior of the separate populations of neurons is shown in response to a 65 dB, 4 kHz stimulus of 100 ms, with a 200Hz saw tooth auditory envelope. This stimulus is shown above the plot. (A) The population of primary-like cells responding to the stimulus. The lines overlayed on the plot show the frequency bands used in the calculation of the metric *γ*, a measure of the prominence of the carrier. The total number of spikes contained in the inner band is divided by the total number of spikes contained by the two outer bands. (B) The Cell-C population without its inhibitory input. The only input is via the excitatory synapses from the primary-like cells shown in (A). The results show that these model Cell-C neurons require coincidence among excitatory inputs to produce an action potential. (C) Cell-C response with the inhibition reinstated. The inhibitory input is delayed by 1.2 ms, and with the excitatory input total delay of 1.6 ms this means that the inhibitory input precedes the excitatory input by 0.4 ms. (D) The *γ* measure as a function of the delay associated with the inhibitory input to the circuit. Note that the total delay of the excitatory input of 1.6 ms is shown by the vertical line. These simulations were completed three times to indicate the low variability from trial to trial; all three resulting data-sets are presented.

To test this hypothesis we then created a more realistic stimulus. An auditory stimulus at 65 dB of 100 ms duration was used to stimulate the circuit. The sound was composed of a carrier of 4 kHz with an envelope of 200Hz. The envelope consisted of sharp onsets with gradual decay, a repeated version of that shown in Fig 7. The primary-like input, as well as the Cell-C neuron responses both without and with inhibition, can be seen in Fig 8A, 8C and 8D, respectively. In Fig 8A, it can be seen that the sound induces a synchronous response across a broad range of ANFs, but an asynchronous response at the carrier frequency. In Fig 8B, it can be seen that, for cells that receive a number of synapses from these ANFs the coincidence in the synchronous response leads to reliable action potentials and the asynchrony at the CF does not lead to significant response. However, if these same cells now receive inhibition that is triggered by broadband response (Fig 8C), then the initial broadband response is suppressed and what follows is enhanced. It appears that the representation of the carrier may be better in this population of cells.

In order to test the hypothesis that the carrier may become more clear under the influence of the inhibition, a metric was used to provide a relative measure of the representation of the carrier in different models. The measure, *γ*, is the total number of spikes produced by a band of 20 Cell-C neurons around the 4 kHz carrier frequency divided by the number of spikes produced by two neighboring bands of 10 neurons each that surround this central band,

$$\begin{array}{c} \gamma =\frac{\sum_{f=F-10}^{F+10}\sum_{t=0}^{N}S_{f,t}}{\sum_{f=F-20}^{F-10}\sum_{t=0}^{N}S_{f,t}+\sum_{f=F+10}^{F+20}\sum_{t=0}^{N}S_{f,t}}, \end{array}$$ (4)

where *S*_*f*, *t*_ represents the spike activity of a Cell-C neuron at frequency *f*.

The metric measures the success of the onset inhibition in suppressing the onset splatter relative to the carrier information. These parameters are illustrated with lines overlayed on Fig 8A. The summations were performed across the whole period of the stimulus centered around the stimulus carrier channel, *F*. A *γ* of 1 indicates no representation of the carrier, with activity in the surround the same as that at the carrier; larger values indicate better representation of the carrier. Values less than 1 indicate that neurons surrounding the carrier are firing at a higher rate than those associated with the carrier.

The neural delay associated with the Cell-B neuron was treated as a free parameter, and its effect on the behavior of the full circuit was examined. As shown in Fig 8D, the delay was systematically varied and *γ* was calculated for each value of the Cell-B neuron delay. This delay adjustment was not meant to reflect any physiological process; it acts as a theoretical test of the function of the circuit. The data very clearly shows a peak in the value of *γ* associated with a Cell-B neuron delay of 1.2 ms. This is an optimum delay at which the population of Cell-C neurons provide a clearer representation of the carrier than the primary-like cells that provided their excitatory input. It should be noted that the total delay of the excitatory input associated with both the cochlear traveling wave delay and the additional compensating delay is approximately 1.6 ms so, at the optimum inhibition delay, the inhibition precedes the excitation, as is known to be the case in the real cells. Examination of the interaction between the excitatory inputs and the inhibitory inputs shows that the splatter present in Fig 8A and Fig 8B is suppressed by the inhibitory influence of the Cell-B neuron and is no longer present in Fig 8C. In addition, the rising portion of the IPSP increases the cell’s propensity to fire. These observations confirm the hypothesis.

It is possible that, unlike a real neuron, the parameters of the model cell used in this study were not optimized for the particular characteristics of the individual inputs from the periphery as well as many other factors. Individual neurons are likely to have specific parameters suited to their own individual environment and functional role [30]. This differentiation process, occurring during development, is likely to be partly environmentally dependent and partly an environmentally independent genetic program for that cell type [31]. So the performance of real cells may be better than this model. Therefore, we investigated whether small changes to the model cell’s parameter values would lead to improvements in the cell’s coding of harmonic content. This was done by tuning the experimentally verified properties of Cell-C neuron IPSPs. Four parameters were subject to alteration: the IPSP amplitude, the rise and decay time constants, and the IPSP delay. A search algorithm was used to improve the value of *γ*. This process is not intended to be strictly optimum but merely to show that, with some adjustment, the function of the circuit can be improved further.

**Fig 9:**
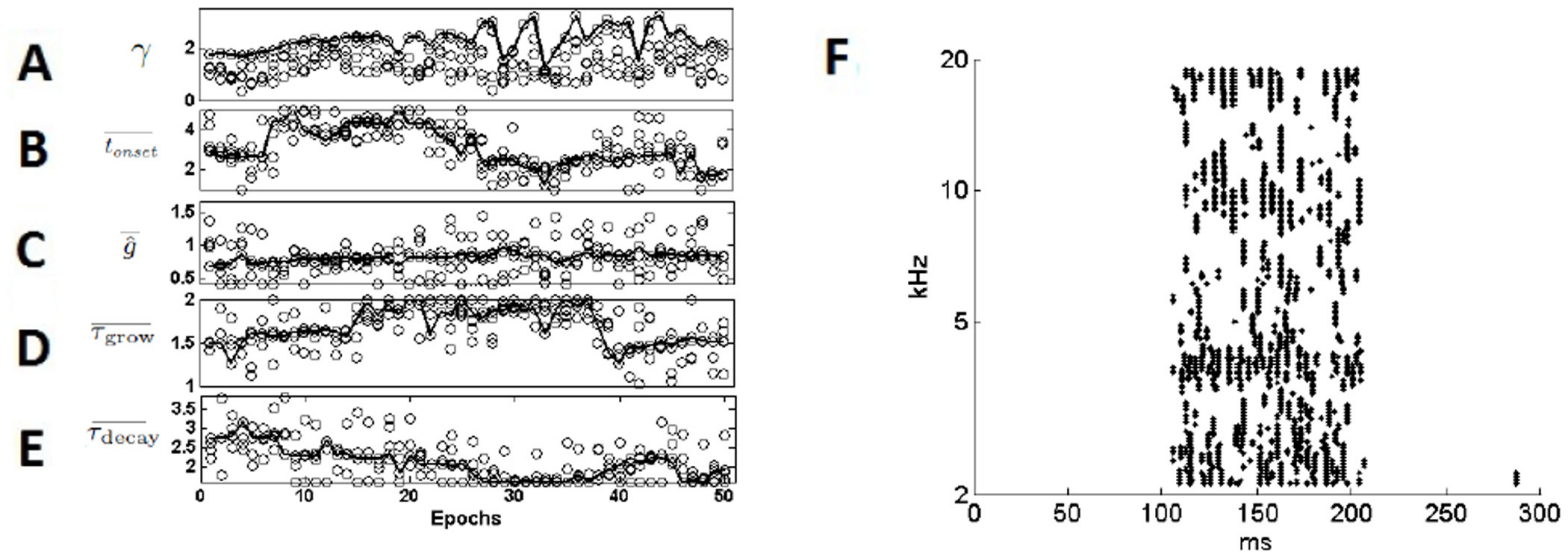
A parameter search used to show improved behaviour of the model with improved representation of the tonal components of the sound. (A) The results of the genetic algorithm showing the evolution of the *γ* as a function of the modeling epoch. The circles indicate the values associated with each “child” of each generation. The lines in each plot indicate the values associated with the “child” that gave the highest *γ*. Generation 36 gave the highest *γ* value, of approximately 3.2. (B, C, D, and E) The evolution of each parameter of the model. The vertical axes show the value of the parameters relative to the experimentally measured values. (F) The response of the population of Cell-C neurons with the adjusted parameters. This can be compared with Fig 8D where directly experimentally derived parameters and produces a lower value of *γ*.

Fig 9A shows the evolution of the *γ* value during the search algorithm; it reached a value three times the original value. Fig 9B–9E show the associated changes in the four parameters of the model. Each variable is quantified as a multiple of the experimentally measured value and plotted as a function of the epoch of the genetic algorithm. Generation 36 gave the best representation of the carrier frequency, as measured by the value of *γ*. At this generation, the IPSP amplitude was large and the IPSPs growth and decay time constants were longer. The IPSP delay was somewhat shorter. None of these changes undermine the apparent mechanism of the circuit. The greater size of the IPSP may be related to an overestimate of the size of the EPSPs in the experimental data, as explained in the experimental methods. Fig 9F shows the behavior of the Cell-C neuron population at generation 36, with the highest *γ* value achieved. It can be seen to provide much clearer representation of the carrier, with a higher spike rate at the carrier frequency than in the surrounding neurons.

### Model Tolerance

**Fig 10:**
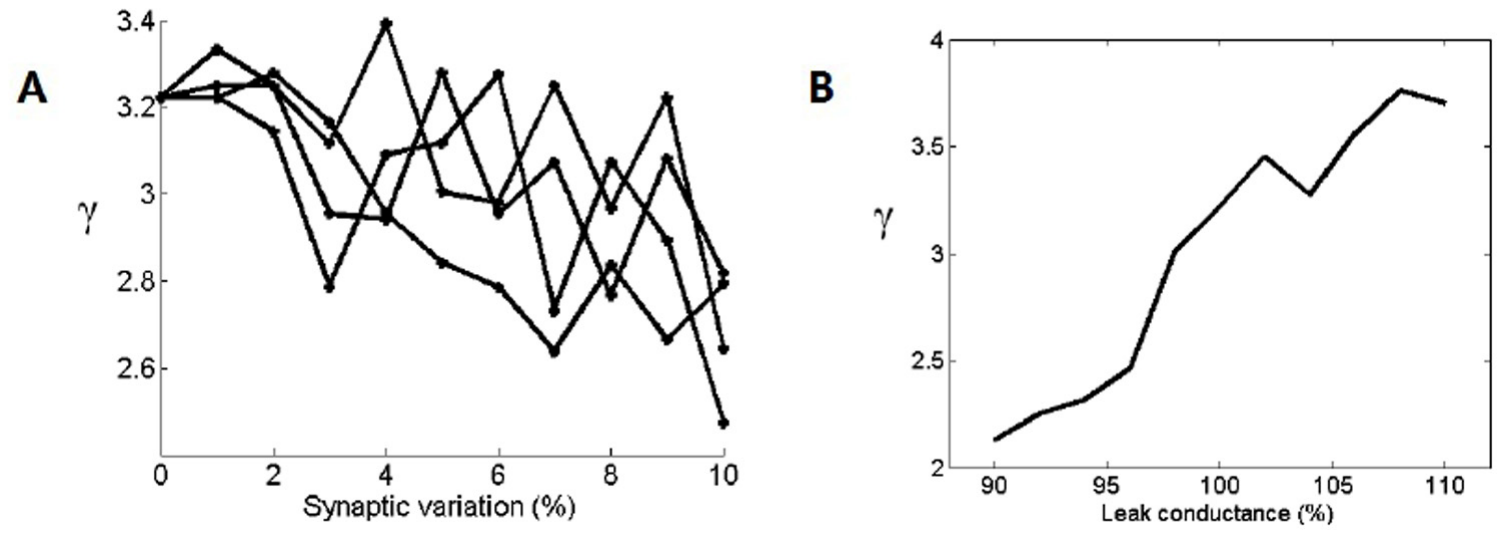
A tolerance analysis of the model to variation in excitatory input strength and membrane leak conductance. (A) The value of *γ* with increasing synaptic variation in the excitatory synapses to the Cell-C neurons. The synaptic weights were set separately and randomly. The resulting *γ* value was found to have high variation and so 4 trials were completed (B) The value of *γ* with variation in the membrane leak conductance 10% above and below the model value.

In the model, it was initially assumed that Cell-C neuron’s synaptic inputs could be set to the same strength. To test the effects of this assumption, the variation in these synapses was systematically increased and the response of the Cell-C neurons were quantified using the *γ* measure. The variation was introduced by using randomized values produced using white noise limited to a certain amplitude. The process was completed a number of times to establish some confidence in the result. These are all shown in Fig 10A. With variation in the synaptic strength within 10% of the mean, the representation of the carrier *γ* was reduced by 15%.

The Cell-C neuron leak conductance was not experimentally constrained and so it was also tested through systematic variation of 10% above and below the model value. In Fig 10B, it can be seen that with an increase of 10% in the leak conductance there was a 17% increase in the value of *γ*. This might indicate that the chosen model conductance was lower than the true value of that in the cell.

## Discussion

The modeling work reported in this study showed that onset inhibition in the auditory brainstem can act as a mechanism to suppress the spectral splatter that occurs at the start of sharp onset sounds. This result was strengthened by four key experimental observations that were used to constrain the model. First, the shape of PSPs in the Cell-C neuron were acquired using a triggered averaging method. Second, the dynamics of the Cell-C neuron onset spike-initiation mechanism was revealed by a phase-space analysis. Third, the spike pattern of the Cell-C neuron in response to the ramped noise stimulus was used to constrain the magnitude of the spike trigger mechanism. Fourth, the number of inputs was constrained by matching the model and experimental membrane voltage displacement during stimulus.

The hypothesis expounded in this paper joins a number of others as an explanation of the purpose of onset inhibition in the mammalian auditory brainstem [9, 10, 5, 11, 13, 12, 2]. It is important to note that these hypotheses are not necessarily mutually exclusive. Different cell populations in the auditory brainstem may utilize onset inhibition for different purposes, and so there may be as many functional roles for onset inhibition as there are different cell populations. It is also possible that different modes of operation may be engaged in different circumstances. For instance, within one neural circuit, it is possible that onset inhibition may delay action potentials under certain conditions and suppress them under different conditions.

In general, splatter suppression may be highly behaviorally relevant. It would be useful where a sound stimulus is produced percussively but where the spectral structure is important for identification. It would be especially useful in cases in which this occurs repeatedly, for example, the vowel sounds produced during human vocalizations.

The method of PSP characterization used in this investigation was inspired by the idea of spike-triggered averaging [16]. It proved to be a very useful analytical method and may find application in a large variety of future experimental investigations. The method may overestimate the amplitude of PSPs when the noise floor impinges on their range of amplitudes. This is because the offset moving average must be set above the noise floor so the accumulating data will not include all of the smallest PSPs, leading to an overestimate of the PSP amplitude. In this study, this qualification applies to the EPSPs but not the IPSPs. This may partly explain why, in Fig 9B, the adapted IPSP seems to increase to 1.5–3 times above its measured value, perhaps compensating for larger EPSPs than are actually present in the real cell.

As noted in the methods section, rather than model the neurons that provide excitatory input to the Cell-C neuron (the Cell-A neuron in Fig 1), it was assumed that these were primary-like cells, which can be modeled as ANFs. This decision may mean that the input spikes were less coherent than in the real system [32], and thus the real VNLL circuit of the brainstem is actually even more efficient than this model suggests. The increased temporal coherence during the splatter is likely to provide greater opportunity for suppression of the splatter by the fast broadband onset inhibition. Additionally, the particular parameters discovered through the search algorithm are also not intended to be perfect. There are many more sophisticated search algorithms available; however, this method successfully produced an improved model, which was all that was required in this case. It was sufficient to show that local parameter variation could improve the circuit’s function.

The phase-plot investigation that was performed (Fig 2B) suggests that Cell-C neurons produce action potentials in response to the rate of change of the membrane voltage exceeding a threshold. This *in vivo* confirmation may be significant as it suggests that, *in vitro*, these cells would show an onset response to step current injection. This fact may help experimentalists to identify these neurons in the future. Additionally, careful examination of the inset of Fig 2B also indicates that an action potential may depend on both the membrane voltage and its rate of change. At more hyperpolarized membrane voltages, the rate of change at which an action potential is produced appears to be higher than at higher membrane voltages. This effect would be an interesting topic of further experimental investigation.

The present investigation suggests that a key role of onset inhibition may be to act in a way that is similar to lateral suppression [33]. Although the mechanisms proposed are somewhat different, both investigations suggest that neural activity that is not associated with the carrier is likely to be reduced by onset inhibition, thus increasing the clarity of the carrier. It should be noted that the model shows that it is only the initial rising phase of the IPSP that suppresses action potentials (Fig 7C). This in turn implies that the relative timing of excitation and inhibition must be very accurate, something that may be difficult to detect in experiments. It may also be possible that the inhibition acts both to suppress particular action potentials and also to enhance the synchrony of those that remain. This would occur by delaying only the earliest occurring action potentials, as suggested in previous studies [9, 10]. To fully capture this effect, a Hodgkin-Huxley model of the cell dynamics should be utilized.

It should be noted that the VNLL of humans is greatly hypertrophied compared to that of cats [17]. In cats, the cells that receive input from octopus cells make up 4% of the nucleus, while in humans they make up 38% of the nucleus. This observation provides indirect evidence that the VNLL may be important for speech processing, a conclusion that is shared with the present investigation.

An expansion of this research could include non-ramped stimuli, stimuli that are more reminiscent of percussive noises, and speech. This approach will allow further detailed evidence to be gathered to test our hypothesis. It would also be instructive to gather data from other areas of the brainstem that receive direct or indirect input from octopus cells. This includes the superior paraolivary nucleus (SPON), which receives projections directly from octopus cells [34, 14, 17], and the inferior colliculus, which receives projections from both the VNLL [35] and the SPON [36]. Since inhibition plays an important role in the circuit, it would be beneficial to use intracellular electrodes. However, a spike triggered average approach may also reveal any suppression of spikes, and this data can be collected extracellularly.

Previous models of octopus cells have used a Hodgkin-Huxley approach [37, 38, 39, 40, 41, 42, 43, 23], with recent models benefitting from data on the dynamics of various ion channel types [44, 45]. The spiking mechanism model used for the octopus cell is a simple, novel approach. This new LIF method may be useful in future investigations into circuits of phasic neurons.

## Conclusion

We created a leaky-integrate-and-fire model of a neural circuit in the mammalian auditory brainstem based on *in vivo* intracellular data gathered from the ventral nucleus of the lateral lemniscus (VNLL). The model revealed that the fast onset inhibition seen in cells of the VNLL can act to suppress the broadband splatter associated with abrupt onsets of sounds. Additionally, it was found that across a population of VNLL neurons, the suppression of the onset splatter enhanced the representation of the harmonic components of speech-like sounds.
